# Regulating community well-being through traditional mourning rituals: Insights from the Luhya People of Kenya

**DOI:** 10.1093/emph/eoaf012

**Published:** 2025-07-09

**Authors:** Stephen Asatsa, Sheina Lew-Levy, Stephen Ngaari Mbugua, Maria Ntaragwe, Wilkister Shanyisa, Elizabeth Gichimu, Jane Nambiri, Jonathan Omuchesi

**Affiliations:** Department of Psychology, The Catholic University of Eastern Africa, Nairobi, Kenya; Department of Psychology, Durham University, Durham, UK; Department of Psychology, The Catholic University of Eastern Africa, Nairobi, Kenya; Department of Psychology, The Catholic University of Eastern Africa, Nairobi, Kenya; Department of Psychology, The Catholic University of Eastern Africa, Nairobi, Kenya; Health Unit, Riara University, Nairobi, Kenya; Department of Psychology, The Catholic University of Eastern Africa, Nairobi, Kenya; Department of Psychology, The Catholic University of Eastern Africa, Nairobi, Kenya

**Keywords:** mourning rituals, cultural evolution, grief therapy, cultural psychology, Indigenous knowledge

## Abstract

**Background and objectives:**

Rituals have been reported to serve as a vital mechanism for expressing grief and fostering communal support worldwide. Despite these benefits, use of rituals in Indigenous communities is threatened by missionization, globalization, and westernization. This study sought to examine the relevance of traditional mourning rituals in community morality and well-being. Anchored in cultural evolutionary theory, the study employed an ethnographic research design.

**Methodology:**

Data were collected from 45 community elders, 30 bereaved adults, 30 bereaved adolescents, and 8 religious leaders through focus group discussions and interviews.

**Results:**

The study established five mourning rituals practiced by the Luhya people, each potentially serving an evolutionary function for community survival and well-being. Our findings show that Luhya traditional mourning rituals play an important role in community well-being, though not all members may benefit equally from these effects.

**Conclusions and implications:**

The study established conflict over rituals with differing viewpoints from religious leaders, cultural leaders, and the western biomedical approach to mental well-being. Yet, the bereaved reported that both Luhya and religious rituals helped them process their grief. To address mental health issues fully, it is important to establish collaboration between western models, religious approaches, and cultural approaches.

## Lay Summary

Rituals are important practices that help people express their grief and provide support within communities around the world yet in most indigenous communities across the world, yet these rituals are being replaced by western biomedical approaches. This study focused on the significance of traditional mourning rituals among the Luhya people of Kenya and how these practices contribute to community morality and well-being. The study interviewed community elders, bereaved adults and adolescents, and religious leaders. The findings revealed that traditional rituals are crucial for the community's health, although not all members experience the same benefits. There were differing opinions about the rituals from religious leaders, cultural leaders, and those who follow western medical approaches to mental health. Despite these conflicts, many bereaved individuals reported that both Luhya and religious rituals helped them cope with their grief. To effectively address mental health issues, the study suggests a need for collaboration between western mental health models, religious practices, and cultural traditions. This approach could create a more comprehensive support system for those experiencing loss.

## INTRODUCTION

Mourning rituals are socially stipulated group conventions performed when a community member has died [1, 2]. These rituals serve as a vital mechanism for expressing grief and fostering communal support, and they are deeply embedded in the cultural fabric of societies worldwide [3]. This perspective highlights the adaptive significance of grief, as it encourages individuals to maintain social bonds even in the face of loss. In rituals, the most ordinary actions and gestures are transformed into symbolic expressions, with their meaning reinforced each time they are performed [4]. The repetitive nature of rituals not only solidifies their significance but also helps individuals navigate their emotional responses to loss. For instance, it has been reported that rituals help individuals appropriately respond to one’s own emotions or the emotions of others [5]. Bereaved individuals who participated in rituals were found to be less sad and processed their grief in a healthier manner compared with those who did not [6]. Rituals have also been found to automatically emerge as a response to psychological and mental health threats, where people in these situations have been reported to develop their own rituals as coping measures to regain control [7]. Populations under intense trauma, such as victims of abuse and palliative care patients, have been found to engage in excessive ritual [8, 9]. This suggests that rituals help individuals adjust to life threatening events. Despite these benefits, in Indigenous communities, the use of rituals is threatened by missionization, globalization, and westernization.

In this paper, we aimed to understand how Kenyan Luhya mourning rituals contribute to community well-being. To do so, we conducted focus groups and interviews with bereaved adults, adolescents, and community leaders. Using a cultural evolutionary framework, we analysed these rituals to identify potentially adaptive features, as well as areas where these rituals may negatively affect well-being. By interviewing religious leaders, we also examined areas of conflict between Luhya, Christian, and Muslim grief rituals. We hope to present an “insider” perspective of rituals to the “outsider” critics, offering a better understanding of the topic from both viewpoints.

### Mental health in Kenya

It is estimated that up to 25% of outpatients and up to 40% of inpatients in health facilities in Kenya suffer from mental health conditions [10]. Suicide rates have been reported to be relatively high in Kenya, with an age-standardized suicide rate of 11.0 per 100 000 population, compared with the global rate of 9.0 per 100 000 population [11]. Furthermore, 9.1% of men in every 100 000 die by suicide compared with 3.2% of women [12]. Yet, mental health remains one of the most underfunded sectors in the Kenyan Ministry of Health, with only 0.01% of the national health budget allocated to mental health services. The country has ∼100 psychiatrists for a population of 54 million, and the numbers of psychologists, social workers, and counselors are very low [13]. Given the mental health status in Kenya, it is important to expand mental health services beyond institutions and professionals to include the community in prevention and treatment. Research on grief and mourning rituals in Kenya is still limited. A study conducted among the Luhya people on effects of restriction of rituals during the COVID pandemic indicated that the disruption had led to psychological distress [14]. This left many community members out of touch with funeral rites, which may lead to complications in grieving [15]. The area of mourning rituals thus requires urgent research. This project is a step toward identifying culturally evolved therapeutic functions of mourning rituals that can help us understand how best to prevent “complicated grief,” where the bereaved remains in a heightened state of mourning, thus keeping them from healing.

### Rituals: benefits and threats

Communal rituals are largely absent from Western psychiatric care despite their widespread prevalence and significant role in healing trauma and grief across cultures. Lang and Kundt [16] suggest that re-examining Western psychiatric models to integrate cooperative practices and community engagement could enhance mental health outcomes. Watson-Jones and Legare [2] emphasize the importance of social connectivity for psychological health, indicating that structured support systems in many non-Western cultures play a crucial role in fostering well-being. Hewlett [17] also highlights the role of communal support and cultural rituals in navigating grief, suggesting that integrating these insights into contemporary healthcare can better address the psychological needs of patients and their families during significant losses.

Yet, scholars agree that many Indigenous cultural practices have been eroded by missionization and colonization, during which missionaries often equated non-Western cultures with degradation, barbarism, ignorance, and darkness [18]. In Africa, this was primarily achieved by persuading Africans to denounce their own cultures, often through coercive means. Similarly, it has been noted that missionaries, in their bid to establish their order, disregarded the established customs of locals, including the significance of rituals [19]. Another study observed that among the Batswana people of Southern Africa, missionaries required Christian converts to wear European clothes and live in European-style houses, effectively stripping them of their cultural identity [20]. In North America, some Indigenous cultural practices were outlawed, with rituals such as ghost dances being banned [21]. The atrocities committed by missionaries against Indigenous cultures have persisted for decades, resulting in a profound loss of cultural heritage. In contemporary African societies, antagonism between cultural practices and the church still exists, manifesting in tensions between traditional beliefs and imposed religious doctrines [22]. Here, we examined the effects of such conflicts on community well-being and on mourning rituals themselves.

### A cultural evolutionary framework

In the present study, we use cultural evolutionary theory to guide us in identifying potentially adaptive features of Luhya mourning rituals. Cultural evolutionary theory posits that humans adapt to their environments primarily through cultural—rather than genetic—adaptations [23]. Through modification and transmission across generations, cultural traits can undergo selection, whereby “certain cultural traits are more likely to be learned and transmitted than others” (p. 484) [24]. Such selection can reflect the mechanism by which it is transmitted or the utility of the trait itself. Rituals have long been viewed as critical mechanisms through which social norms and religious beliefs are transmitted, leading to cultural continuity [25, 26]. As a site of one-to-many transmission, ritual involves highly effective forms of communication which can result in rapid dissemination [27–30]. Rituals may also create a structured environment that enhances focus, engagement, and retention of information [31–33]. Specifically, the repetitive and symbolic nature of rituals fosters a sense of community and belonging among participants, which in turn can facilitate deeper cognitive processing [32]. This framework guides us to focusing on “who participates” in different Luhya mourning rituals, and “the content” of these rituals, to identify potentially adaptive features. We also explore how Christian and Muslim religious leaders perceive traditional Luhya rituals. Such insights can shed light on how inter-group contact shapes culture change, a growing topic of interests in cultural evolution [34].

### The present study

In total, we identified 25 rituals among the Luhya. For this paper, we focused on five rituals (see Table 1) related to unnatural death (suicide, murder) and gender norms (fertility, fidelity) because: (i) these rituals evoke knowledge related to community survival and reproduction, with direct fitness consequences for individual and community well-being, and (ii) they are a site where power dynamics are negotiated and affirmed, thus allowing us to consider the uneven well-being effects that these rituals may produce.

**Table 1:** Section of code book and themes

CODES	Themes	Files	Frequency
• Beating the corpse for suicide deaths	Beating the corpse	6	12
• Beating the corpse			
• Burial for unnatural deaths	Retaliation rituals	17	38
• Burial with sword and torch			
• Khusola (attacking murders family)			
• Burial outside the compound	Shaming rituals	10	27
• Creating gate for the deceased			
• Burial at night			
• Burial of childless people	Fertility rituals	3	5
• Piercing manhood			
• Putting on the deceased hat or coat during burial	Family preservation rituals	10	20
• Carrying the deceased portrait and looking into the grave			
• Khukhala makhola (sexual Cleansing)			
• Re-marrying			

## METHODOLOGY

This study was conducted among the Luhya community of Kenya, the second largest tribe in the country, making up about 14% of Kenya’s total population [35]. The tribe primarily relies on agriculture as its main economic activity, although many Luhya individuals are also professionals in various sectors of the economy. We worked with the five most populous and traditional clans: Bashitsyula, Bukusu, Tiriki, Banyala, and Batsotso. The study was approved by the Africa International University Institutional Ethical Review Board, number ISERC/EXT085/2023. A study license (NACOSTI/P/23/25017) was obtained from the National Commission for Science, Technology, and Innovation. Further clearance was obtained from each of the three county governments where the Luhya clans reside. The researchers also obtained permission from community elders and other gatekeepers before engaging community members in the study. Full information about the study was disclosed to participants before seeking their consent to participate. Separate consent was obtained to audio record the interviews. For adolescents, parental consent and individual assent were obtained before their participation in the study. Due to the emotive nature of the study, psychologists were hired to debrief participants to address any possible emotional reactions anticipated after the study.

We conducted focus group discussions with community elders, bereaved adults, and bereaved adolescents, as well as in-depth interviews with religious leaders. Focus groups are an ideal way to elicit thoughts, feelings, and beliefs from community experts through discussion among themselves and the moderator [36]. Each focus group consisted of 8–10 participants from homogeneous members of the community to respect the power structures within this community. Data were collected through five focus groups for elders, four for bereaved adults, four for bereaved adolescents, and eight in-depth interviews with religious leaders. The study sample included community elders (67% male, 33% female) aged 65–91 years; bereaved adults (60% male, 40% female) aged 35–75 years; and bereaved adolescents (55% male, 45% female) aged 12–21 years. It should be noted that this is a highly patriarchal community, and the status of males in society is still viewed as more esteemed compared with that of women. During recruitment, the researchers invited both men and women to participate but observed that slightly fewer women than men signed up. It was also noted during focus group discussions that women appeared passive, with men dominating the conversations. This issue was mitigated by encouraging women to speak when researchers noted male domination.

Community elders were interviewed to understand their perceptions regarding the benefits of traditional Luhya mourning rituals and the ongoing threats to these practices. Bereaved adults and adolescents were interviewed to discuss their roles in the mourning rituals and their perceptions of these rituals. Christian and Islamic religious leaders were interviewed to understand their beliefs regarding traditional Luhya mourning rituals and to identify conflicts and convergences between Christian, Muslim, and Luhya practices. The sample size balanced the time needed to conduct in-depth ethnographic interviews on one hand and the number of participants needed to achieve thematic saturation on the other [37].

Interviews and focus group discussions were recorded using audio recorders, backed up with field notes. Each team consisted of two interviewers, two technical staff to operate the recorders, two note-takers, and two observers. This was done to ensure that data were captured by more than one source, thereby assuring the credibility and dependability of the collected data. Data were processed using NVivo Version 14. The data were transcribed and coded using open and inductive coding. The codes were categorized and grouped, from which themes were later generated using thematic analysis. After generating the themes, community validation was conducted. This involved returning to the field with a summary of the themes and meeting the participants in their focus groups to check if the analysed data reflected what they had said during data collection. This process generated additional narratives that helped enhance the preliminary data. In addition to focus group discussions and interviews, the research team also visited funerals, bereaved families, and elders to directly observe mourning rituals.

## RESULTS

Our direct observations suggest that grief rituals among the Luhya unfold in three stages: preburial, during burial and postburial periods. Grief intensity varies over time: the different stages of rituals help to address the changing intensity of emotions and attachment to the deceased over time. The pre-burial period rituals focused on gathering of family and friend, preparing the body and deciding on a burial site. The burial period rituals focused on ceremonial observance with ceremonies such as funerals, night vigils, and other community gatherings. Postburial rituals focused on commemoration by engaging in activities that remember the deceased. We now expand on five rituals: (i) shaming the deceased, (ii) beating the corpse for suicide deaths, (iii) retaliation rituals for homicide deaths, (iv) family preservation rituals, and (v) fertility-related rituals.

### Shaming the deceased

For suicide deaths, the burial is conducted at night, with only the maternal uncles and mature male members of the deceased allowed to participate. Maternal uncles are preferred for these kinds of burials since they usually have no blood relations with the paternal side of the family and hence cannot transmit the spirit of suicide through blood kinship after interaction with suicide corpse. Women and children are not allowed to participate. In some clans, the burial is conducted at the exact place where the death occurred. The process of burial is deliberately made unpleasant with many ceremonies prohibited, as it is believed that suicide victims do not deserve to be given decent burial as this may appease the spirit of suicide hence encouraging repetitive suicide deaths in the family. Suicide victims are not washed or dressed well like the bodies of other natural deaths. During the burial, lights are switched off in the entire compound. Women and children ordered to get indoors with only men beyond childbearing ages allowed to be outside to witness the burial. The coffin is not procedurally lowered into the grave, but it is thrown in without caring whether it lands on the wrong side. The deceased is cursed as the burial goes on and ordered to go completely and never to appear to anyone in the family. The grave of the suicide victim is finished differently. It is not supposed to end with a heap of soil like other graves but should be flattened with grass planted on it. This is to make the person forgotten as fast as possible. The suicide object or tree (if the person hangs on a tree) is destroyed, uprooted, and burnt. The destruction of the tree or object associated with a suicide is viewed as a way to cleanse the site and prevent further contamination or negative spiritual influences. Participants said:

**Bukusu Elder 3:** For somebody who has committed suicide, the time that I’m buried even my own family is not allowed to be closer only my maternal uncles will bury me at night and the family will take out a sacrifice and give it to the uncle, that is what happened long ago, then they bury me at night; the mother is not allowed there, children are not allowed there, and even youth are not allowed there, people from my family are not allowed there. It’s only my uncles that are allowed. There, they go and talk to the deceased as they lower him in the grave, then they come back.

**Bashitsyula Elder 1:** Such a person should be forgotten as fast as possible. No family wants to continue remembering such a shameful death. The pain of holding on this death for long yet it is already shameful can devastate the family.

### Beating the corpse

This ritual is performed for people who die of suicide. The community believes in the sanctity of life and highly condemns the act of terminating one’s own life or someone else’s life. Those who die of suicide are seen as an abomination to the community. To keep away the spirit of the deceased from causing similar deaths, the deceased is usually beaten and buried in a humiliating manner. The burial happens at night and is conducted by members of a different community, usually that of the deceased’s maternal uncles. However, religious leaders (both Muslim and Christian) from the Luhya community oppose this ritual, terming it as demonic and ungodly:

**Butsotso Pastor:** I do not support this practice. Beating a corpse to send away spirits is demonic. The bible is clear on how spirits are sent away. In the Christian, this can be achieved through prayer.

**Sheikh:** Islam teaches respecting the body of the deceased. Beating the body of the dead is disrespectful.

Even though this ritual appears scary, some participants reported to have felt better after participating in it. For instance, one adult said:

**Bunyala bereaved Widow 4:** Definitely, it brings healing and it releases emotions because at that particular time you have many questions why this person had to kill himself.

This implies that engaging in this ritual creates room for catharsis, where the grieving members of the community vent out negative emotions. Participants reported different experiences they had with this ritual:

**Tiriki Elder 1:** Here somebody who hangs himself using rope, that one is beaten up, beating him up shows that he goes with that so that he does not haunt the living. The first person who gets him is the one who beats him and not just with the stick, you properly beat him saying something, “When you go, go forever do not come back and start harassing people, because you did that by yourself.”

Some other members endorsed both religious and traditional rituals related to murder and suicide, while others recommend adherence to religious rituals only:

**Bereaved Adult:** Personally, all the rituals make me happy, because the tradition follows all the things that the dead did, he goes with them. And the Christian preach for him so that God opens the door for him to go and stay with him and therefore personally to me, all those things they are good.

### Retaliation rituals

Retaliation rituals directly avenge perpetrators of a community member’s murder. For murder victims, burial is conducted at night by the maternal uncles of the deceased. Women and children are not allowed to witness the burial process or view the body. When bringing the body back home, it does not go through the main gate; a new gate for that purpose has to be created through the fence. Once the body goes through the gate, is not used by other people but closed in a ceremony that involves slaughtering a black sheep. This is done to keep the spirit of murder away from the family. For all unnatural deaths, children cannot be named after these victims, as it is believed that they could die in the same way. An elder said:

**Bashitsyula Elder 5:** And if the person was killed, we cannot name him, No newborn is named after anyone killed in another town, killed while stealing or committed suicide. We seal (funga kabisa) the name. Even a person who has been killed, we do not name children after him…No. Maybe if you call a child that name, he will be killed too.

Retaliation can be done by the community (*Okhusola*) if the murderer is known or by the deceased (burial with weapons) if the murderer is unknown.

#### Revenge by the surviving relatives (Okhusola)

If the murderer is known and a member of the community, clan members of the deceased organize retaliatory responses against the perpetrator where they attack his home and force the murder out. The perpetrator’s houses are destroyed, their land sold, and the proceeds used to resettle the person in a different community. During the raid, the raiders are not allowed to kill the perpetrator. The perpetrator is never allowed to live among the community where he/she committed the murder:

**Bashitsyula Bereaved adolescent 5**: A crowd of rowdy men singing war songs and shouting ran towards his compound from different directions. In a few minutes his banana plantation was destroyed his animals raided and the houses were on fire... We have never seen the murder again in our village. (**Was this fair)?** Yes I felt nice that justice for my uncle had been served and it feels very relieving.

#### Revenge by the dead

Where the murderer is unknown, the victim is buried facing down, with a torch, knife, and a special grass called “lusubanjilu” placed in their hands. This is done to enable the deceased to “go after” and attack those who murdered them. During the burial, the person is spoken to and instructed to go after the killers and exterminate them. If he is a man, he is reminded to remain a man even in death and go after his killers. It is believed that after this kind of burial, the spirit of the deceased will seek justice for the victim, where it will hunt and kill all those involved in the death. A participant said:

**Batsotso Bereaved adult 2:** Those buried with a torch or a stick in their hands, it is the same as I said: When I’m on the road and somebody kills me and so my family does not know who killed me, during burial, they give me a torch so that I go seek them by myself and kill them. During burial they say, “we have given you this torch, go follow those people and destroy them. It is so that they also be found the way they found you.”

Religious leaders opposed this ritual terming it ungodly. One Pastor said:

**Bukusu Religious leader 2:** Revenge belongs to the Lord. We cannot encourage our members to advance hatred to the deceased or perpetrators. We preach forgiveness and not revenge.

The traditional burial rituals and practices surrounding unnatural deaths among the Luhya reflect a complex interplay of cultural beliefs, spiritual worldviews, and symbolic meanings. These practices serve to protect the living, seek justice for the deceased, and maintain the social and spiritual order within the community. While some of these customs may be considered unorthodox or even unethical from a western perspective, they nonetheless hold deep significance within the cultural context in which they are practiced:

**Butsotso Bereaved Widow 9:** Eeeh, I tell you that home, for now when you see them it is completely destroyed. **(They spoke at the same time).** All those who participated in killing my husband all died. They got finished, because of that herb and torch he was buried with. (**Oooh, it means they died)?** Yes, it finished. Everybody who participated in killing him died. He took the instructions he was given on going after the killers and avenging his own death seriously. **(Laughter)**.

The expression of happiness in describing the effect of the ritual on perpetrators indicates the relieving effect it has on the family members of the deceased.

### Family preservation rituals

Family preservation rituals encourage public showcasing of marital fidelity in the case of spousal death. The community places great importance on fidelity to marriage and family. In the case of the death of a spouse, there are certain rituals that are designed to reward faithfulness. When a husband has died, the widow is expected to put on the deceased husband’s coat or hat and dance around the grave while carrying the deceased’s portrait or his walking stick. This is a symbol of her devotion to the man, and it shows that she has lived her life faithful to the man. The elders assert that if a woman was ever unfaithful to the man, she should never perform this ritual. Otherwise, she will die a few days after the burial of her husband. During burials, the elders indicated that when they see a widow who has not performed this ritual, they conclude that she was not faithful to her husband. Unfaithful spouses are also not supposed to look directly into the grave of their spouse during burial or even touch the soil usually thrown in the grave during burial. The elders emphasize that these rituals and restrictions are not limited to women, as they also apply to unfaithful men. Widows who participated in this ritual reported feeling respected and encouraged by the community:

**Bukusu Elder 2**: Even unfaithful men will not look at the wife’s grave fully; other men will come and take him away.

Elders maintain that the onus of faithfulness and adherence to these practices is primarily on the woman. Furthermore, it is clear that this ritual applies more to widows as widowers are never expected to wear the deceased’s wife coat or hold their walking stick:

**Bukusu Elder 3**: aaaaah Men do not carry portraits. They move all over.

**Bukusu Elder 1**: Now let me ask you. From the time you were born (pointing to younger people in the room) have you seen a man carry the portrait? (Pause) A man is like a cock.

It was also reported that widows who wish to remarry should not perform this ritual:

**Bashitsyula Elder 1**: The women used to wear the deceased husband’s clothes to indicate that they were faithful to the man.

**Bashitsyula Elder 3**: Even such an unfaithful woman does not sit near the dead husband’s body and during the burial; she will step back from the grave. (Uuhmmm) Some may pretend to look inside the grave and yet they are looking aside. When burying when you see her moving backwards…just know she was unfaithful (Uuhmmmm) she looks aside, infidelity?

**Banyala bereaved widow 3**: I felt so encouraged and respected after carrying his portrait and wearing his coat. I felt like the community absolved me from any unspoken blame. It was a message to other family members that I was faithful. However, this should also be enforced for widowers. It is unfair to women, if applied selectively.

### Fertility-related rituals

Childless women are buried outside the compound, often behind the house. This is done to symbolically represent the absence of a child, as the lack of offspring is seen as a significant social and spiritual deficiency. During the burial, a guard or other object is placed with female deceased to represent a “baby,” accompanied by specific rituals and incantations to prevent the “childless spirit” from following or influencing the living. Male childless individuals are buried with a thorn pierced on their manhood or buttocks. One elder describes a man who dies without a child as stupid who must be buried in the most disrespectful manner to chase away the spirit of childlessness. The Luhya burial rituals for the unmarried and childless individuals reflect the community’s deep-rooted cultural beliefs and social norms surrounding marital status and procreation. The segregated burial practices serve to reinforce the societal expectations and importance placed on marriage and childbearing, ensuring the continued preservation of the community’s social structures and values. The burial of the childless outside the compound, or with a symbolic representation of a child, is rooted in the belief that the lack of offspring disrupts the ancestral lineage and continuity of the family:

**Bukusu bereaved widow 4**: I feel bad when I see childless women treated this way because it was not their fault. I think culture should treat such women with respect during burial.

**Bukusu Elder 6:** (With gestures) But don’t you think this will maintain the spirit of childlessness in our community)?

This ritual appears controversial with female members of the community appearing to oppose it, while males support it. The ritual is viewed as discriminative against innocent members of the community who die without a child.

## DISCUSSION

Ethnopsychologists have long recognized significant cultural variation in distress and psychiatric illness [38]. Specifically, Kirmayer (p. 149) [39] notes that “culture influences the experience, expression, course and outcome of mental health problems, help-seeking and the response to health promotion, prevention or treatment interventions.” Furthermore, Indigenous healing practices may represent culturally adaptive strategies promoting better mental health outcomes across communities [40]. Guided by cultural evolutionary theory, we contribute to this body of work by examining who participates in different Luhya mourning rituals, and the content of these rituals, to identify potentially adaptive features.

We found that the burial of suicide victims is conducted away from the community, usually at night, with other death-related rituals suspended. This is done to prevent the spirit of the deceased from causing similar deaths. This ritual approach to suicide may reflect a culturally evolved strategy for limiting the transmission of suicidal behavior [41–43]. Indeed, research has found a significant association between exposure to suicidal behavior among adolescent peers and subsequent adolescent suicide attempts [42]. Furthermore, suicide deaths bring stigma and shame to family and friends of the deceased [44] with suicide bereavement reported to be among the most stigmatizing sudden loss [45]. Shaming rituals may generate cathartic experiences, hence serving as coping mechanisms for the affected community members by shifting their frustration to the deceased.

One area of future research we hope to explore regards the role of power and domination in the cause of suicide, and the rituals associated with suicide, among the Luhya. Previous research suggests that power and conflict play a substantial, and likely causal, role in evoking suicidal behavior [46–48]. For example, among Aguaruna, women committed suicide as a means of punishing their social partners (husbands, fathers) for deteriorating social relationships [49]. In South Africa, Niehaus [50] argues that women die of suicide to protest their subordination in a male dominated society, while men die of suicide in response to lost domination. Other scholars have reported higher suicide rates among highly patriarchal societies with women and low status men being more vulnerable [51, 52]. In Kenya in general, and anecdotally, among the Luhya, men are more likely to commit suicide than women—in future work, we hope to further understand these gendered dynamics. In addition to potentially preventing the spread of suicide, shaming rituals may represent cultural tools for maintaining power within the community while presenting the deceased as the weaker link [48]. Shaming rituals may also prevent individuals from seeking help, leading to further harm and stigmatization of the already socially vulnerable suicide survivors. Future work will investigate how participation in shaming rituals affects individual mental health following the suicide death of a loved one, and how this covaries with social status within the broader community.

We found restrictions on participation in the burial of murder victims. Studies have reported that exposure to dead bodies from war is a risk factor for Posttraumatic Stress Disorder [53]. Given that most murder deaths are traumatic, the restrictions imposed on certain categories of community members may protect those viewed as vulnerable from psychological trauma. It is noted that the community hands over the responsibility of avenging the unknown murderer themselves to the deceased. This could prevent escalating retaliatory killings of perceived perpetrators. Burial of murder and suicide victims is conducted by strangers to the community, potentially building cross-community alliances. Overall, retaliation and burial rituals for murder cases may work towards de-escalation which could promote both community and individual well-being.

While weddings are considered fertility and fidelity rituals across cultures [54], our findings suggest that burials can also display and reinforce reproductive goals and strategies (to other women, not necessarily the widow or widower) at a time of high emotional arousal. Public showcasing of marital fidelity may enhance family cohesion [55, 56] and at the same time transmit the virtue of fidelity to other men and women attending the funeral. Comparing the fidelity rituals for men and women among the Luhya, it appears that women’s rituals are more conspicuous. This implies that it is easier for the mourners to identify a faithful or unfaithful woman compared with men. The fidelity ritual may thus also serve to reinforce gendered social hierarchy [57]. Declining to participate in this ritual can create shame and stigma for the bereaved spouse, signaling a lack of fidelity to the deceased which may negatively impact their individual well-being. This is also the case with fertility-related rituals, where childless members of the community are treated differently in death. In as much as treating the childless and unmarried differently at death aims to transmit procreation and family values to community members, it should be noted that the deceased might not have had control over their situation. This could trigger distress among other childless community members attending the funeral. In our future work, we plan to focus on how gender-specific fertility and fidelity norms are transmitted during rituals, as well as how these articulate with changing gender norms in broader Kenyan society [58].

Our findings established a cultural conflict for some rituals within the Luhya community. As seen in the practices surrounding the deceased who die by suicide and the revenge rituals for those who die by murder, the Christian and Muslim religious leaders insist on the agency belonging to God, while the Luhya community members insist the agency belongs to the community and to the dead. This echoes studies implying an increase in conflict between Christianity and African culture in the nineteenth century [59]. The clash between Christianity and cultural practices has led to a serious identity crisis among South Africans, which can lead to self-hatred [60]. Where religion discourages participation in rituals but does not offer alternative coping mechanisms, the well-being of the bereaved might be compromised. However, some rituals are blending: we participated in several rituals where church leaders prayed before the traditional rituals began. For instance, before the community engages in wailing, a religious leader has to pray for the deceased. This collaboration promotes cooperation between community and religious leaders, two groups that have a history of antagonism. In future studies, we will examine how community members innovate upon mourning rituals (e.g. by blending elements) and how such reconfigured ceremonies affect the well-being of the bereaved.

## CONCLUSION

Our findings show that Luhya traditional mourning rituals play an important role in community well-being, though not all members may benefit equally from these effects. The antagonistic-cooperation nature of Christianity, Islam, and Luhya traditional beliefs suggests that conflict arises over rituals with differing viewpoints regarding the agency of the dead. Yet, the bereaved reported that both Luhya and Christian/Muslim rituals helped them process their grief. Overlooking culture and context, and overreliance on a narrow pharmacological paradigm that is mostly associated with the western biomedical model, has been reported to degrade the quality of mental health care [61]. The model maintains a reductionist approach of symptom control, leaving out crucial agents such as patient’s religion, socioeconomic status, interpersonal relationships, and cultural meaning of illness, [62] which affects uptake of treatment [63]. To address mental health issues fully, it is important to establish collaboration between western models and cultural approaches. Our findings point to several fruitful avenues for research in cultural evolution: in our future work, we hope to more quantitatively explore what, how, and from whom knowledge is transmitted during mourning rituals. Using surveys, we also hope to better understand how participation in traditional vs. Christian or Muslim rituals affects Luhya mental health, especially in cases of traumatic death (suicide, murder). In terms of practical implications, and considering the lack of mental health support available in Kenya, local communities could be included in the prevention and treatment of mental illness. By bridging individual psychiatric care with communal rituals, practitioners may discover new pathways to healing that acknowledge the vital importance of community and shared experiences in promoting emotional stability and resilience. Training local counselors on the potential benefits of traditional Luhya mourning rituals while sensitizing them to potential harms for socially vulnerable individuals may, in some cases, represent a culturally appropriate strategy for improving mental health.

### Declaration of generative AI and AI-assisted technologies in the writing process

During the preparation of this manuscript, we utilized Monica, an AI language model developed by Open AI, for grammar editing and enhancing the clarity of the article. After using this tool, we reviewed and edited the content as needed and therefore we take full responsibility for the content of the publication.
